# Estimating Surface Area in Early Hominins

**DOI:** 10.1371/journal.pone.0016107

**Published:** 2011-01-13

**Authors:** Alan Cross, Mark Collard

**Affiliations:** 1 Laboratory of Human Evolutionary Studies, Department of Archaeology, Simon Fraser University, Burnaby, British Columbia, Canada; 2 Department of Anthropology, University of Missouri, Columbia, Missouri, United States of America; University of Illinois at Champaign-Urbana, United States of America

## Abstract

Height and weight-based methods of estimating surface area have played an important role in the development of the current consensus regarding the role of thermoregulation in human evolution. However, such methods may not be reliable when applied to early hominins because their limb proportions differ markedly from those of humans. Here, we report a study in which this possibility was evaluated by comparing surface area estimates generated with the best-known height and weight-based method to estimates generated with a method that is sensitive to proportional differences. We found that the two methods yield indistinguishable estimates when applied to taxa whose limb proportions are similar to those of humans, but significantly different results when applied to taxa whose proportions differ from those of humans. We also found that the discrepancy between the estimates generated by the two methods is almost entirely attributable to inter-taxa differences in limb proportions. One corollary of these findings is that we need to reassess hypotheses about the role of thermoregulation in human evolution that have been developed with the aid of height and weight-based methods of estimating body surface area. Another is that we need to use other methods in future work on fossil hominin body surface areas.

## Introduction

Thermoregulation is generally accepted to have been an important factor in human evolution. For example, thermoregulation is thought to have been involved in both the transition from quadrupedalism to bipedalism around 5–7 Ma, and the transition from the australopith body form to the *Homo erectus* form about 1.9 Ma [1]–[3]. Likewise, several Neanderthal characteristics are considered to be adaptations to glacial conditions [4], [5].

In order to understand the role played by thermoregulation in human evolution it is necessary to obtain accurate estimates of the body surface area of fossil hominins. Because skin absorbs and loses heat, its quantity and distribution have a major impact on the thermoregulatory abilities of an organism. All other things being equal, an organism with a high surface area to body mass ratio will lose heat more rapidly than an organism with a lower surface area to body mass ratio.

To date, two ways of estimating fossil hominin surface areas have been utilized by palaeoanthropologists. One is to create a three-dimensional anatomical model of the species of interest and measured the model's surface area directly [e.g. 2], [ 5]. The other is to employ one of the equations that have been developed by medical researchers to predict surface area in living humans from weight and height [e.g. 2], [ 3].

Here, we report a study that focused on the latter approach. The goal of the study was to assess the accuracy of height and weight-based methods when applied to early hominins. A number of the height and weight-based equations in question have been validated for living humans [6]. So there is reason to believe that the estimates they yield for fossil hominins with body proportions that are similar to those of humans are accurate. However, their application to early hominins is a different matter. None of the equations is capable of taking into account limb proportion differences between taxa. Yet it is clear from the fossil record that early hominin limb proportions were markedly different from those of humans [7]. As such, it is possible the estimates the equations yield for the early hominins are inaccurate.

The research protocol we employed in the study entailed comparing surface area estimates generated with the most widely used height and weight-based method to estimates generated with a method that is sensitive to body proportion differences. The height and weight-based method we employed was developed by Dubois and Dubois [8]. The other weight and height-based methods differ from Dubois and Dubois' method (hereinafter the DDM) and from each other simply in the coefficients and exponents employed. None of them is capable of taking into account limb proportion differences among taxa. As such, we reasoned that, if we found the DDM to be inaccurate, the other weight and height-based methods could be assumed to be inaccurate too. We began by confirming that the two methods are equally accurate when applied to living humans. We then ensured that the additional assumptions required to apply the methods to skeletal specimens do not result in different estimates. Lastly, we used both methods to generate estimates for several hominin species, including the early hominins *Ardipithecus ramidus* and *Australopithecus afarensis*, and compared the estimates.

## Materials and Methods

In the DDM, surface area is calculated as follows: Surface area (cm^2^)  = 0.007184*H^0.725^*W^0.425^. H in this equation is height (cm), and W is weight (kg). The body proportion-sensitive method we utilized was outlined by Cross et al. [9]. Cross et al.'s [9] method (hereinafter the CCNM) models the body as 14 cylinders. The length (L) and circumference (C) of each body segment are measured (in cm), and these values are used to solve the formula for the surface area of a cylinder minus its ends: Surface area (cm^2^)  =  ΣCL. Subsequently, the surface areas of all segments are summed to determine total surface area.

To confirm the DDM and CCNM are equally accurate for living humans, we applied both methods to data for 26 variables recorded on seven adult males, and then compared the two sets of estimates. The variables included stature, weight, and the length and circumference of the head and neck. Lengths and upper and lower circumferences were recorded for each of the other segments. Mid-segment circumferences were also calculated for the trunk and lower legs. All limb measurements were recorded on the right side of the body. The mean stature, weight and segment dimensions for the living human sample are presented in Table S1. The data were collected at the University of Western Ontario with the approval of that institution's ethics review board (Review #11120E) and the written informed consent of the volunteers. The estimates were compared with the paired t-test (p≤0.05).

To ensure the additional assumptions required to apply the DDM and CCNM to skeletal specimens do not result in different estimates, we applied both methods to data from four human skeletal samples—Afro-Americans, Euro-Americans, Egyptians and Inuit. These human populations were selected because of their representativeness of the latitudinal variation displayed in the fossil hominin sample. We obtained mean values for the length of the long bones for the Afro-Americans, Euro-American, Egyptian and Inuit samples from Trinkaus [10], and used population-appropriate equations to generate estimates of stature and body mass. Details of these samples are given in Table S2.

To apply the CCNM, we estimated the surface area of each limb segment by multiplying the length of the relevant bone by the mean surface area per cm for the limb segment (a proxy for mean circumference) in question in the living human sample. The surface areas of the other body segments were then estimated by summing the limb segment surface areas, dividing the resulting figure by the mean percentage of total surface area represented by the limb segments in the living human sample, and then multiplying the quotient by the mean percentage of total surface area represented by the other body segments in the living human sample. Again, the estimates were compared with the paired t-test (p≤0.05).

We applied the DDM and CCNM to two early hominins—*Au. afarensis* and *Ar. ramidus*—and three later hominins—*Homo neanderthalensis*, African *Homo erectus* and Asian *Homo erectus*. In addition, we applied the DDM and the CCNM to *Homo floresiensis*. The limb proportions of *H. neanderthalensis* and *H. erectus* are similar to those of humans. In contrast, the legs of *Ar. ramidus* and *Au. afarensis* are much shorter relative to their arms than are those of humans [7]. Although *H. floresiensis* may have survived into the Holocene, its limb proportions are clearly more similar to the limb proportions of the early hominins than to the limb proportions of the hominin taxa it overlaps with temporally, *H. erectus*, *H. neanderthalensis* and *H. sapiens*. Hence, we consider it to be an early hominin.

Details of the specimens we used are given in the Table S2, along with the sources for estimates of stature, mass, and long bone lengths. We applied the CCNM to Neanderthals and *H. erectus* in the same way as we applied it to the four human skeletal samples. The same procedure was also used for *Ar. ramidus*, *Au. afarensis* and *H. floresiensis* except we employed surface area per cm values that were intermediate between the living human sample and values for the chimpanzee calculated from Crompton et al. [11] (see Table S3). We followed this course of action because the three taxa in question were considerably more robust than humans [12]–[14]. Once again, the estimates were compared with the paired t-test (p≤0.05).

Lastly, to examine the effect of modeling the nonhuman-like fossil hominins as more robust than the human-like ones, we repeated the comparison of DDM and CCNM estimates for the six fossil hominin taxa after calculating the total surface areas of *Ar. ramidus*, *Au. afarensis* and *H. floresiensis* using the same segment circumference values as were used for the other hominin taxa.

## Results

The mean difference between the DDM and CCNM estimates for the living human sample is only 3% (Table 1). This is not significant according to the paired t-test (p = 0.07).

**Table 1:** Percentage difference between DDM- and CCNM-derived surface area estimates (cm^2^) for living humans, human skeletal samples and fossil hominins.

Taxon	DDM	CCNM	%Diff.
Living human mean	19,359	18,939	3
Afro-American skeletons	17,754	18,946	6
Euro-American skeletons	17,405	17,813	2
Egyptian skeletons	16,835	18,172	7
Inuit skeletons	16,922	16,258	4
*Homo neanderthalensis*	18,008	18,055	0
Asian *Homo erectus*	14,483	15,514	7
African *Homo erectus*	19,587	21,296	8
*Homo floresiensis*	9,686	12,233	21
*Australopithecus afarensis*	9,337	12,370	25
*Ardipithecus ramidus*	12,325	14,048	12

Most of the differences between the DDM and CCNM estimates for the four human skeletal samples are similar in magnitude to the differences identified for the living humans (Table 1). When the surface area estimates for the human skeletal samples are combined with those for the living humans, the difference between the DDM and CNNM is not significant (p = 0.81).

The differences between the DDM and CCNM estimates obtained for the fossil hominins vary considerably (Table 1). The smallest difference between the two estimates was for the Neanderthals (<1%), while a 25% discrepancy was identified for the *Au. afarensis* estimates. In contrast to the situation when the DDM and CCNM estimates for the human samples were compared, the two estimates for the fossil hominins are significantly different (p = 0.01).

The analysis carried out to evaluate the effect of modeling the nonhuman-like fossil hominins as more robust than the human-like fossil hominins also returned a statistically significant difference between the DDM and CCNM estimates (p = 0.04). Thus, the statistical difference between the CCNM and the DDM obtained in the third analysis is not the product of modeling the three nonhuman-like hominins differently to account for their greater robusticity.

## Discussion

The first two analyses demonstrate that the DDM and CCNM yield indistinguishable surface area estimates when applied to humans, regardless of whether the sample comprises just live individuals or both live individuals and skeletal specimens. The third and fourth analyses show that the DDM and CCNM yield significantly different estimates when applied to fossil hominins with human-like body proportions and fossil hominins whose body proportions differ markedly from those of humans. Given that the DDM ignores limb proportion differences while the CCNM takes such differences into account, these findings suggest that the DDM is not accurate for estimating surface area in fossil hominins with limb proportions that differ from those of humans.

As explained in the Introduction, we focused on the DDM because it is the most widely used height and weight-based method, and we reasoned that, since the other height and weight-based methods only differ from the DDM in the coefficients and exponents employed, if the DDM was found to be inaccurate, the other height and weight-based methods could be assumed to be inaccurate too. However, other height and weight-based methods have been claimed to be more accurate for estimating surface area in *H. sapiens* than the DDM [5], [15], [16]. So, it is possible that we would have obtained different results if we had used one of the other methods. To investigate this possibility, we repeated the third analysis after estimating surface areas with the height and weight-based method outlined by Gehan and George [17]. We employed this method because it has been argued to be more accurate than the DDM [15], [16] and was recently employed in a study dealing with Neanderthal thermoregulation [5].

The differences between the CCNM estimates and the estimates yielded by Gehan and George's [17] method were insignificant for the living human sample (p = 0.09). This was also true when the four human skeletal samples were added to the sample (p = 0.48). In contrast, a significant difference was identified between the CCNM estimates and the estimates yielded by the Gehan and George [17] method for the fossil hominins (p = 0.02). This is the same pattern as we obtained with the DDM. Thus, it appears that our finding that the DDM and CCNM yield significantly different estimates when applied to fossil hominins with body proportions that differ from those of humans is not peculiar to the DDM. As we had assumed, other height and weight-based methods also produce inaccurate surface area estimates when applied to fossil taxa whose body proportions differ from those of humans.

While the results of our analyses are consistent with the idea that the height and weight based methods are inaccurate when applied to fossil hominins with nonhuman-like limb proportions they do not conclusively demonstrate that such is the case. They show the height and weight based methods are inaccurate when applied to some fossil hominins, but it is possible the inaccuracy is caused by something other than the limb proportions of *Ar. ramidus*, *Au. afarensis* and *H. floresiensis*.

To evaluate this possibility, we carried out two sets of supplementary analyses. In the first, we used the paired t-test to compare the DDM and CCNM estimates for the four human skeletal samples and the fossil hominins with human-like body proportions (*H. neanderthalensis*, and African and Asian *H. erectus*). We then added the DDM and CCNM estimates for the other three fossil hominins to the sample and ran another t-test. In the first t-test the DDM and CCNM estimates were indistinguishable (p = 0.06), while in the second t-test they were significantly different (p = 0.01). Given that the DDM and CCNM estimates only diverged when *Ar. ramidus*, *Au. afarensis* and *H. floresiensis* were added to the sample, this analysis supports the idea that the DDM's inaccuracy is in fact driven by hominins with limb proportions that differ from those of humans.

**Figure 1:**
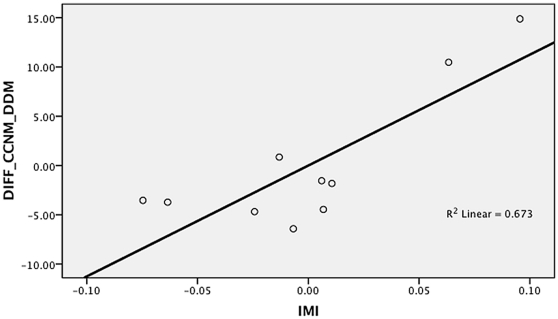
Partial regression plot of intermembral index (IMI) against percent difference between surface area estimates yielded by DDM and CCNM (DIFF_CCNM_DDM).

**Figure 2:**
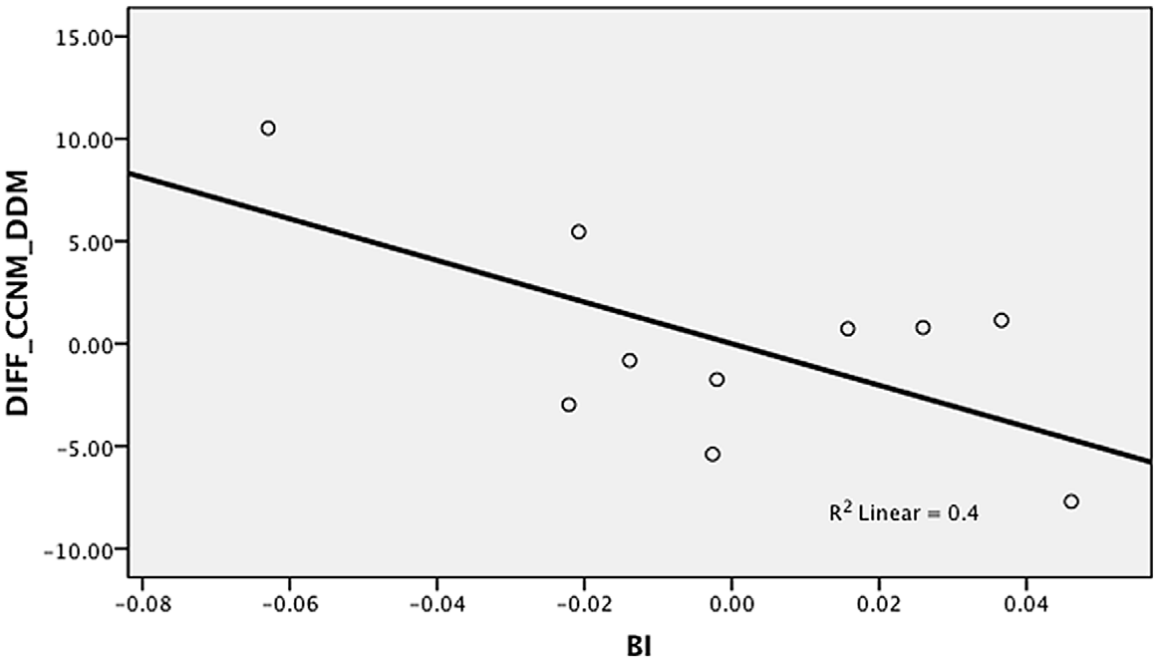
Partial regression plot of brachial index (BI) against percent difference between surface area estimates yielded by DDM and CCNM (DIFF_CCNM_DDM).

**Figure 3:**
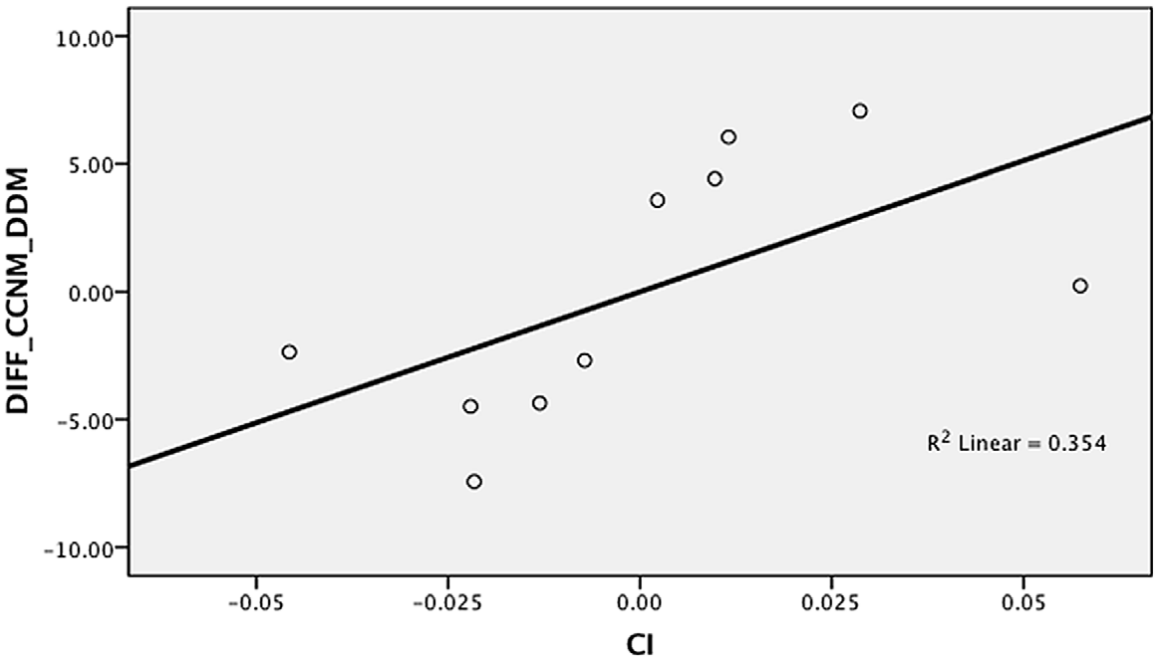
Partial regression plot of crural index (CI) against percent difference between surface area estimates yielded by DDM and CCNM (DIFF_CCNM_DDM).

In the second set of supplementary analyses, we used regression to measure the impact of limb proportion differences on the accuracy of the DDM. All the human skeletal samples and fossil hominins were included in the analysis. The percentage difference between the DDM and CCNM estimates was used as the dependant variable, and the brachial, crural and intermembral indices as the independent variables. The analysis indicated that both the intermembral index (Fig. 1) and the brachial index (Fig. 2) have a major impact on the accuracy of the DDM. The partial correlation coefficient for the intermembral index was r = 0.820, while for the brachial index r = −0.633. In contrast, the crural index (Fig. 3) did not to have a significant impact on accuracy of the DDM. The partial correlation coefficient for the crural index was only r = 0.595. When all three independent variables were included in a multiple regression, a strong significant result was obtained (r = 0.858, p = 0.036) and an independent effect was identified for each predictor. Given that the three limb proportion indices explain 86% of the variation in the differences between the DDM and CCNM estimates, the results of the regression analysis support the idea that the DDM yielded inaccurate estimates when applied to the fossil hominin sample because the latter included taxa with limb proportions that differ from those of humans.

It appears, then, that our concerns about the application of height and weight-based methods of estimating surface area to fossil hominins with limb proportions that differ from those of humans are valid. The analyses we have reported here demonstrate not only that height and weight-based methods yield significantly lower estimates of surface area for such hominins than a method that takes into account body proportion differences, but also that the differences between the height and weight-based estimates and the estimates yielded by the body proportion-sensitive method are largely due to the limb proportions of the hominins in question.

These findings are, of course, only as good as the data used in the analyses. The main cause of concern in this regard is the bone length, stature and body mass estimates for the fossil hominins that we obtained from the literature. Because standard errors are not available for many of these estimates [e.g. 13], [ 18], we were forced to treat them as if they were absolute values. This undoubtedly means our results are less secure than is desirable. However, previous attempts to estimate fossil hominin surface areas have been similarly constrained [2], [3], [5]. So, our results are as secure as those of any other study in this area.

The results of our study have at least two important implications. One is that parts of the current consensus regarding the role played by thermoregulation in human evolution need to be reassessed. Perhaps the most obvious of these is the hypothesis that the thermal demands of inhabiting hot, dry, open environments played a role in selecting against early hominin limb proportions because they would have been disadvantageous in later, larger-bodied hominins. This hypothesis was proposed by Wheeler [2] and subsequently supported by Ruff [3]. Both of these authors argued in favour of the hypothesis on the basis of the results of analyses in which they used the DDM to estimate the surface area of an early hominin, *A. afarensis*. Thus, given that our study indicates the DDM and other height and weight-based methods yield inaccurate estimates of surface area when applied to early hominins, there is a need to re-test the hypothesis taking into account the limb proportion differences between early hominins and later hominins.

Another important implication of our results concerns the way we estimate fossil hominin body surface areas in the future. Given that height and weight-based methods yield inaccurate estimates for early hominins, and that the first five millions years of human evolution was dominated by early hominins, height and weight-based methods are clearly unlikely to help us understand the role of thermoregulation in human evolution. As such, we need to use other methods when estimating fossil hominin body surface areas.

If we disregard height and weight-based methods, which method should we use to of estimate fossil hominin body surface areas? At the moment, there are two options. One is to create three-dimensional anatomical models of the species of interest and measure the models' surface areas directly. The other is to use the CCNM. As long as every assumption in the construction process is clearly documented so that the model can be replicated, there is nothing wrong with constructing anatomical models. However, the time and effort required to build such models seem unwarranted when there now exists a much simpler method capable of producing accurate estimates of body surface area in only a couple of minutes. Unless there are additional reasons for constructing a scale model, the ease of implementation of the CCNM make it the preferable option for acquiring surface area estimates of hominins.

In sum, the goal of the study reported here was to assess the accuracy of height and weight-based methods of estimating body surface area when applied to early hominins. We were concerned that the limb proportions of the early hominins, which differ substantially from those of humans, might cause the body surface area estimates generated with height and weight-based methods to be inaccurate. The results of the study indicate that our concern was warranted. We found that height and weight-based methods are accurate when applied to humans and hominins with human-like limb proportions, but inaccurate when applied to hominins with limb proportions that differ from those of humans. This finding means that we need to reassess the parts of the current consensus regarding the role played by thermoregulation in human evolution that are based on the results of applications of height and weight-based methods of estimating body surface area. It also means that future work on fossil hominin body surface area should avoid height and weight-based methods of estimating surface area, and utilize instead methods that are capable of taking into account inter-taxa differences in limb proportions.

## Supporting Information

Table S1 **Measured values and estimates for living human sample.** Values are means for seven adult males.
(DOC)

Table S2 **Measured values and estimates for human skeletal samples and fossil hominin taxa.**
(DOC)

Table S3 **Data for** ***Pan troglodytes*** **used to estimate surface area per unit length values and total surface area for** ***Homo floresiensis*** **,** ***Australopithecus afarensis*** **and** ***Ardipithecus ramidus*** **.** Values are from Crompton et al. (1).
(DOC)
