# Supplementary material for: Estimating Surface Area in Early Hominins
Source: PLoS One. 2011 Jan 13;6(1):e16107. doi: 10.1371/journal.pone.0016107 (PMC3020943; doi:10.1371/journal.pone.0016107)
Supplement: Table S1 — Measured values and estimates for living human sample. Values are means for seven adult males. (DOC) [file pone.0016107.s001.doc]

**Table S1 for Cross & Collard’s**

**‘Estimating surface area in early hominins’**

**Table S1. Measured values and estimates for living human sample. Values are means for seven adult males.**

| **Variable** | **Value** |
| --- | --- |
| Head length | 21 cm |
| Head circumference | 57 cm |
| Neck length | 12 cm |
| Neck circumference | 37 cm |
| Upper arm length | 33 cm |
| Upper arm upper circumference | 33 cm |
| Upper arm lower circumference | 26 cm |
| Lower arm length | 27 cm |
| Lower arm upper circumference | 26 cm |
| Lower arm lower circumference | 17 cm |
| Upper leg length | 31 cm |
| Upper leg upper circumference | 60 cm |
| Upper leg lower circumference | 36 cm |
| Lower leg length | 43 cm |
| Lower leg upper circumference | 35 cm |
| Lower leg middle circumference | 37 cm |
| Lower leg lower circumference | 25 cm |
| Stature | 178 cm |
| Weight | 76kg |
| Total surface area | 18,939 cm^2^ |
| Surface area of limbs | 9,166 cm^2^ |

All values have been rounded off.

**Raw data and estimates for living human sample**

Means of the measured values and estimates for the seven living humans are presented in Table S1. The volunteers were all male, and were aged between 22 and 26. Total surface area was calculated with Cross et al.’s (1) method. This method models the body as 14 cylinders. The length (L) and circumference (C) of each body segment is measured, and these values are used to solve the formula for the surface area of a cylinder minus its ends:

Surface area (cm^2^) = ∑CL

Subsequently, the surface areas of all segments are summed to determine total surface area. The surface area of the limbs was obtained by calculating the surface area of each limb segment using the formula for a cylinder minus its ends, and summing the resulting values.

**References**

1. Cross A, Collard M, Nelson A (2008) Body segment differences in surface area, skin temperatures and 3D displacement and the estimation of heat balance during locomotion in hominins.  PLoS ONE 3, e2464
