# Supplementary material for: Estimating Surface Area in Early Hominins
Source: PLoS One. 2011 Jan 13;6(1):e16107. doi: 10.1371/journal.pone.0016107 (PMC3020943; doi:10.1371/journal.pone.0016107)
Supplement: Table S2 — Measured values and estimates for human skeletal samples and fossil hominin taxa. (DOC) [file pone.0016107.s002.doc]

**Table S2 for Cross & Collard’s**

**‘Estimating surface area in early hominins’**

**Table S2. Measured values and estimates for human skeletal samples and fossil hominin taxa**

|  | Afro-American skeletal sample | Euro-American skeletal; sample | Inuit skeletal sample | Egyptian skeletal sample | *Homo neanderthalensi*s | Asian *Homo erectus* | African *Homo erectus* | *Homo floresiensis* | *Australopithecus afarensis* | *Ardipithecus ramidus* |
| --- | --- | --- | --- | --- | --- | --- | --- | --- | --- | --- |
| Upper arm length (cm) | 34 | 33 | 30 | 33 | 34 | 30 | 40 | 24 | 25 | 28 |
| Lower arm length (cm) | 26 | 25 | 23 | 27 | 26 | 24 | 33 | 21 | 19 | 26 |
| Upper leg length (cm) | 47 | 45 | 41 | 45 | 46 | 39 | 50 | 28 | 29 | 31 |
| Lower leg length (cm) | 40 | 37 | 33 | 39 | 37 | 31 | 45 | 24 | 25 | 26 |
| Upper arm surface area per unit length (cm^2^/cm) | 29 | 29 | 29 | 29 | 29 | 29 | 29 | 32 | 32 | 32 |
| Lower arm surface area per unit length (cm^2^/cm) | 22 | 22 | 22 | 22 | 22 | 22 | 22 | 21 | 21 | 21 |
| Upper leg surface area per unit length(cm^2^/cm) | 49 | 49 | 49 | 49 | 49 | 49 | 49 | 49 | 49 | 49 |
| Lower leg surface area per unit length(cm^2^/cm) | 33 | 33 | 33 | 33 | 33 | 33 | 33 | 25 | 25 | 25 |
| Total surface area (cm^2^) | 18,946 | 17,813 | 16,258 | 18,172 | 18,055 | 15,514 | 21,296 | 12,233 | 14,325 | 16,143 |
| Stature (cm) | 172 | 170 | 159 | 167 | 171 | 154 | 185 | 106 | 107 | 121 |
| Weight (kg) | 66 | 64 | 67 | 61 | 69 | 50 | 73 | 36 | 33 | 51 |

All values have been rounded off.

**Raw data and estimates for human skeletal samples and fossil hominin taxa**

The measured values and estimates used to calculate the surface areas of the four human skeletal samples and the six fossil hominin taxa are presented in Table S2. The sources of the measured values and estimates are as follows:

Afro-American skeletal sample All values are adult male means. Humerus, femur and tibia lengths were obtained from Trinkaus (1). Following Haeusler and McHenry (2), ulna length was estimated by adding 5% to the radius length given by Trinkaus (1). Mean segment circumference values were derived from the living human sample. In the living human sample ca. 27% of the proximal femur is situated within the trunk segment (as determined by palpation of the greater trochanter). Accordingly, this amount was subtracted from the femora before the surface area of the upper leg was estimated. Total surface area was calculated by summing the limb segment areas, dividing the resulting figure by the percentage of total surface area represented by the limbs in the living human sample (48.4%), and then multiplying the quotient by 100. Stature was estimated from femur length using Trotter and Gleser’s (3) equation for Blacks: 2.10(Femur Length) + 72.22. Weight was calculated from stature with Ruff and Walker’s (4) formula: Weight = 0.888(Stature) – 87.1. This formula was developed from a worldwide sample of 40 populations.

Euro-American skeletal sample All values are adult male means. Humerus, femur and tibia lengths were obtained from Trinkaus (1). Following Haeusler and McHenry (2), ulna length was estimated by adding 5% to the radius length given by Trinkaus (1). Mean segment circumference values and total surface area were calculated in the same manner as they were for the Yugoslavian skeletal sample. Stature was estimated from femur length using Trotter and Gleser’s (3) equation for Whites: 2.32(Femur Length) + 65.53. Weight was calculated from stature with the same formula that was used to calculate weight for the Afro-American sample (i.e. Weight = 0.888[Stature] – 87.1).

Inuit skeletal sample All values are adult male means. Humerus, femur and tibia lengths were the Alaskan Eskimo/Inuit values presented in Trinkaus (1). Following Haeusler and McHenry (2), ulna length was estimated by adding 5% to the radius length given by Trinkaus (1). Mean segment circumference values and total surface area were calculated in the same manner as they were for the Yugoslavian and Euro-American skeletal samples. Stature for the Inuit sample was estimated using the Eskimo/Inuit femur:stature ratio of 25.72 from Feldesman and Fountain (5) and is equal to the mean stature for male “Eskimos” provided by Ruff (6). Because the Inuit have been argued to carry more weight per unit of femur length (e.g. 7) estimates derived from femur length may underestimate weight in Inuit. We therefore used the mean bi-iliac breath for Eskimo/Inuit (Bi-Iliac Breadth = 29.7cm) presented in Ruff (6) and then calculated body weight using Ruff et al.’s (8) equation for males: Weight = 0.422(Stature) + 3.126(Bi-Iliac Breadth) - 92.9. We used this equation because the sample from which it is derived includes a number of high latitude populations.

Egyptian skeletal sample All values are adult male means. Humerus, femur and tibia lengths were obtained from Trinkaus (1). Following Haeusler and McHenry (2), ulna length was estimated by adding 5% to the radius length given by Trinkaus (1). Mean segment circumference values and total surface area were calculated in the same manner as they were for the other human skeletal samples. Stature was calculated using Raxter et al.’s (9) equation for the femur: 2.257(Femur Length) + 63.93. We used this equation because it was developed for use with ancient Egyptians. Weight was calculated from stature using the same formula that was used to calculate weight for the Afro-American and Euro-American samples (i.e. Weight = 0.888[Stature] – 87.1).

*Homo neanderthalensis* Values are for La Ferrassie 1. Humerus, femur and tibia lengths were obtained from Trinkaus (1). Following Haeusler and McHenry (2), ulna length was estimated by adding 5% to the radius length given by Trinkaus (1). Surface area per unit length values and total surface area were calculated in the same manner as they were for the human skeletal samples. Stature was taken from Churchill (10) who summed the estimated height of various elements and then added an additional 9cm to account for soft tissue and match a previous estimate for this individual. Weight was calculated by employing the formula provided by Ruff and Walker (4) (Weight = 0.888[Stature] – 87.1) and then increasing muscle mass by 12.9%. Our weight estimate of 65kg is identical to that cited by Sorenson and Leonard (11) for LF1. Churchill (10) estimated LF1’s body weight to be 85kg but this was based on a hypothetical bi-iliac breadth estimate (i.e. the pelvis for this individual was not recovered). Churchill (10) estimated that Neanderthal muscle attachment sites were approximately 12.9% larger than his cold-adapted modern human reference sample. If we assume that this translates into 12.9% greater muscle weight and that male human muscle represents approximately 41.5% of total body weight (12), then increasing muscle mass by 12.9% would produce an approximate body weight of 68.5kg.

Asian *Homo erectus* Values are for the large adult specimen from Dmanisi reported by Lordkipanidze et al (13). Weight, stature, and the lengths of the humerus, femur and tibia taken from Lordkipanidze et al (13). These authors estimated weight with equations for femur, humerus, tibia, and first metatarsal developed from a mixed sample of adult great ape and human specimens. They estimated stature from humerus length using equations for human juveniles. Ulna length was estimated from the length of the humerus using the equation provided by Häusler (14). Surface area per unit length values and total surface area were calculated in the same manner as they were for the human skeletal samples and *H. neanderthalensis*.

African *Homo erectus* Values are for KNM-WT 15000. Weight, stature and long bone lengths are the estimated adult values presented in Ruff and Walker (4). These authors report that adult stature was estimated using estimated adult long bone lengths from the juvenile skeletal remains and inserting those into equations derived from warm-adapted adult modern human reference populations. They report that weight was estimated using their estimates of adult stature and bi-iliac breadth, inserting those data in equations for a variety of reference samples, and averaging the results. Surface area per unit length values and total surface area were calculated in the same manner as they were for the human skeletal samples, *H. neanderthalensis* and Asian *H. erectus*.

*Homo floresiensis* Values are for LB1. Long bone lengths from Brown et al. (15) and Moorwood et al. (16). Stature and weight from Brown et al. (15). Brown et al.‘s (15) value for the stature of LB1 is the average of estimates derived from several equations. The equations in question were developed with femur length data from human pygmy populations and a range of different regression techniques. Brown et al. (15) made several estimates of body weight using their stature estimate and femur cross sectional area. In view of the robusticity of LB1, we used the one derived from a mixed African ape and human reference sample. To estimate the segment surface area per unit length values for *H. floresiensis*, we followed the procedure described for the human skeletal samples and the other fossil hominin taxa except we used values that are intermediate between those for the living human sample and a *Pan* *troglodytes* specimen reported by Crompton et al. (17). We adopted this course of action because *H. floresiensis* was considerably more robust than humans (16). The relevant data for the *P. troglodytes* specimen are presented in Table S3. As with the human skeletal samples and the other fossil hominin taxa, 27% was subtracted from the femora before the surface area of the upper leg was estimated. Total surface area was calculated by summing the limb segment areas, dividing the resulting figure 45.7%, and then multiplying the quotient by 100. 45.7% is the midpoint between the percentage of total surface area represented by the limbs in the living human sample and the equivalent figure for the *P. troglodytes* specimen.

*Australopithecus afarensis* Values are for AL 288-1. Femur length was obtained from McHenry and Berger (18). Humerus and ulna lengths were taken from Hausler (14). Tibia length was obtained from Schmid (19). Stature was taken from Ruff (20). Weight is the average of the female weight estimates derived from hindlimb joint size given by McHenry (21). Surface area per unit length values and total surface area were calculated in the same manner as those for *H. floresiensis*.

*Ardipithecus ramidus* Weight, stature and long bone lengths were obtained from Lovejoy et al. (22). Weight was estimated by Lovejoy et al. (22) using regression equations developed from a variety of reference samples, including all anthropoids, female anthropoids, great apes and humans, and female great apes and humans. Lovejoy et al. (22) do not explain how they estimated stature. Surface area per unit length values and total surface area were calculated in the same manner as those for *H. floresiensis* and *Au. afarensis*.

**References**

1. Trinkaus E (1981) Neanderthal limb proportions and cold adaptation. In Stringer CB (ed) Aspects of Human Evolution. London: Taylor and Francis, pp. 187-224

2. Haeusler M, McHenry HM (2004) Body proportions of *Homo habilis* reviewed. J Hum Evol 46:433–65

3. Trotter, ML and Gleser G. 1958. A re-evaluation of stature based on measurements taken during life and of long bones after death. Am J Phys Anthropol 16:79-123.

4. Ruff CB, Walker A (1993) Body size and body shape. In: Walker A, Leakey RE (eds) The Nariokotome Homo erectus Skeleton. Cambridge: Harvard University Press, pp.234-265

5. Feldsman MR, Fountain RL (1996) “Race” specificity and the femur/stature ratio. Am J Phys Anthropol 100: 207-224

6. Ruff CB (1994) Morphological adaptations to climate in modern and fossil hominids. Ybk Phys Anthropol 37:65-107

7. Shepard RJ, Hatcher J, Rode A (1973) On the body composition of the Eskimo. Europ J Appl Physiol 32(1): 3-15

8. Ruff CB, Niskanen M, Junno J-A, Jamison P (2005) Body mass prediction from stature and bi-iliac breadth in two high latitude populations, with application to earlier higher latitude humans. J Hum Evol 48: 381-392

9. Raxter MH, Ruff CB, Azab A, Erfan M, Soliman M, El-Sawaf A (2008) Stature estimation in ancient Egyptians: A new technique based on anatomical reconstruction of stature. Am J Phys Anthropol 136:147-155

10. Churchill SE (2008) Bioenergetic perspectives on Neandertal thermoregulatory and activity budgets. In: Harvati K, Harrison T (eds) Neanderthals Revisited: New Approaches and Perspectives. New York: Springer, pp. 113-133

11. Sorenson MV, Leonard WR (2001) Neandertal energetics and foraging efficiency. J Hum Evol 40:483-495

12. Aiello LC, Wheeler P (1995) The expensive-tissue hypothesis: The brain and digestive system in human and primate evolution. Curr Anthropol 36(2): 199-221

13. Lordkipanidze D, Jashashvili T, Vekua A, Ponce de León MS, Zollikofer CPE, Rightmire GP, Pontzer H, Ferring R, Oms O, Tappen M, Bukhsianidze M, Agusti J, Kahlke R, Kiladze G, Martinez-Navarro B, Mouskhelishvili A, Nioradze M, Rook L (2007) Postcranial evidence from early *Homo* from Dmanisi, Georgia. Nature 449:305-310

14. Häusler M (2001) New insights into the locomotion of *Australopithecus africanus*: Implications of the partial skeleton of STW 431 (Sterkfontein, South Africa). Ph.D. Dissertation, Universität Zürich.

15. Brown P, Sutikna T, Moorwood MJ. Soejuno RP, Jatmiko, Wayhu Saptomo E, Rokus Awe Due (2004) A new small-bodied hominin from the Late Pleistocene of Flores, Indonesia. Nature 431:1055-1061

16. Moorwood MJ, Brown P, Jatmiko, Sutikna T, Wahyu Saptomo E, Westaway KE, Rokus Awe Due, Roberts RG, Maeda T, Wasisto S, Djubiantono T (2005) Further evidence for small-bodied hominins from the Late Pleistocene of Flores, Indonesia. Nature 437:1012-1017

17. Crompton RH, Li Y, Alexander R McN, Wang W, Gunther MM (1996) Segment inertial properties of primates: New techniques for laboratory and field studies of locomotion. Am J Phys Anthropol 99:47-570

18. McHenry HM, Berger LR (1998) Body proportions in *Australopithecus afarensis* and *A. africanus* and the origin of the genus *Homo*. J Hum Evol 35:1-22

19. Schmid, P (1983) Eine Rekonstruktion des Skelettes von AL 288-1 (Hadar) und deren Konsequenzen. Folia Primatol 40:283-306

20. Ruff CB (1991) Climate and body shape in hominid evolution. J Hum Evol.

21: 81-105

21. McHenry HM (1992) Body size and proportions in early hominids. Am J Phys Anthropol 87:407-431

22. Lovejoy CO, Suwa G, Simpson SW, Matternes JH, White TD (2009) The great divides: *Ardipithecus ramidus* reveals the postcrania of our last common ancestors with African apes. Science 326(73):100-106
