# Supplementary material for: Estimating Surface Area in Early Hominins
Source: PLoS One. 2011 Jan 13;6(1):e16107. doi: 10.1371/journal.pone.0016107 (PMC3020943; doi:10.1371/journal.pone.0016107)
Supplement: Table S3 — Data for Pan troglodytes used to estimate surface area per unit length values and total surface area for Homo floresiensis , Australopithecus afarensis and Ardipithecus ramidus . Values are from Crompton et al. (1). (DOC) [file pone.0016107.s003.doc]

**Table S3 for Cross & Collard’s**

**‘Estimating surface area in early hominins’**

**Table S3. Data for *Pan troglodytes* used to estimate surface area per unit length values and total surface area for *Homo floresiensis*, *Australopithecus afarensis* and *Ardipithecus ramidus*. Values are from Crompton et al. (1).**

| **Variable** | **Value** |
| --- | --- |
| Upper arm length (cm) | 29 |
| Lower arm length (cm) | 29 |
| Upper leg length (cm) | 29 |
| Lower leg length (cm) | 24 |
| Upper arm circumference (cm) | 35 |
| Lower arm circumference (cm) | 20 |
| Upper leg circumference (cm) | 49 |
| Lower leg circumference (cm) | 17 |
| Surface area of limbs (cm^2^) | 6,843 |
| Total surface area (cm^2^) | 16,143 |

All values have been rounded off.

**References**

1. Crompton RH, Li Y, Alexander R McN, Wang W, Gunther MM (1996) Segment inertial properties of primates: New techniques for laboratory and field studies of locomotion. Am J Phys Anthropol 99:47-570
